# Supplementary material for: Nutritionally Enriched Muffins from Roselle Calyx Extract Using Response Surface Methodology
Source: Foods. 2022 Dec 8;11(24):3982. doi: 10.3390/foods11243982 (PMC9778146; doi:10.3390/foods11243982)
Supplement: Supplementary file 1 [file foods-11-03982-s001.zip › foods-2043587-supplementary.pdf]

Table S1: Sensory respondents' score of different muffin formulation resulting from RSM.

Formulation	Aroma	Colour & Appearance	Body & Texture	Taste & Flavour	Overall Acceptability
01	6.10 ± 1.42	6.68 ± 1.56	6.36 ± 1.54	5.23 ± 1.97	5.7 ± 1.78
02	5.23 ± 1.50	5.60 ± 1.24	6.63 ± 0.92	5.70 ± 1.48	6.01 ± 1.11
03	7.06 ± 0.69	7.10 ± 1.44	6.93 ± 1.11	7.60 ± 1.19	7.53 ± 0.97
04	6.43 ± 0.93	6.90 ± 1.12	6.60 ± 1.28	6.40 ± 1.47	6.65 ± 1.25
05	6.60 ± 0.85	6.93 ± 0.86	7.23 ± 0.97	6.50 ± 1.40	6.53 ± 1.27
06	6.20 ± 1.12	6.11 ± 1.04	6.46 ± 1.00	6.26 ± 1.04	6.26 ± 0.94
07	6.43 ± 1.07	7.10 ± 1.12	6.73 ± 1.28	7.60 ± 1.22	6.78 ± 0.99
08	6.73 ± 1.14	7.23 ± 1.04	7.13 ± 1.27	6.36 ± 1.51	6.66 ± 1.24
09	6.50 ± 1.30	6.83 ± 1.17	6.63 ± 1.09	6.73 ± 1.28	6.70 ± 1.08
10	6.73 ± 0.98	7.13 ± 1.07	6.88 ± 0.63	7.26 ± 1.08	7.30 ± 0.83
11	7.20 ± 0.80	7.50 ± 0.90	7.23 ± 0.72	7.73 ± 0.73	7.80 ± 0.80
12	7.30 ± 0.79	7.73 ± 0.73	7.00 ± 0.69	7.60 ± 0.77	7.60 ± 0.77
13	6.33 ± 0.99	6.53 ± 1.30	7.20 ± 1.03	7.10 ± 1.12	7.10 ± 0.99
14	6.26 ± 1.25	6.33 ± 1.06	6.96 ± 1.15	6.23 ± 1.33	6.38 ± 1.32
15	6.46 ± 1.10	6.90 ± 0.99	7.23 ± 0.85	6.46 ± 1.35	6.53 ± 0.93
16	6.10 ± 1.18	6.16 ± 1.08	6.46 ± 1.16	6.10 ± 1.44	6.20 ± 1.12
17	7.06 ± 0.69	7.73 ± 0.69	6.76 ± 0.67	7.63 ± 0.76	7.56 ± 0.62

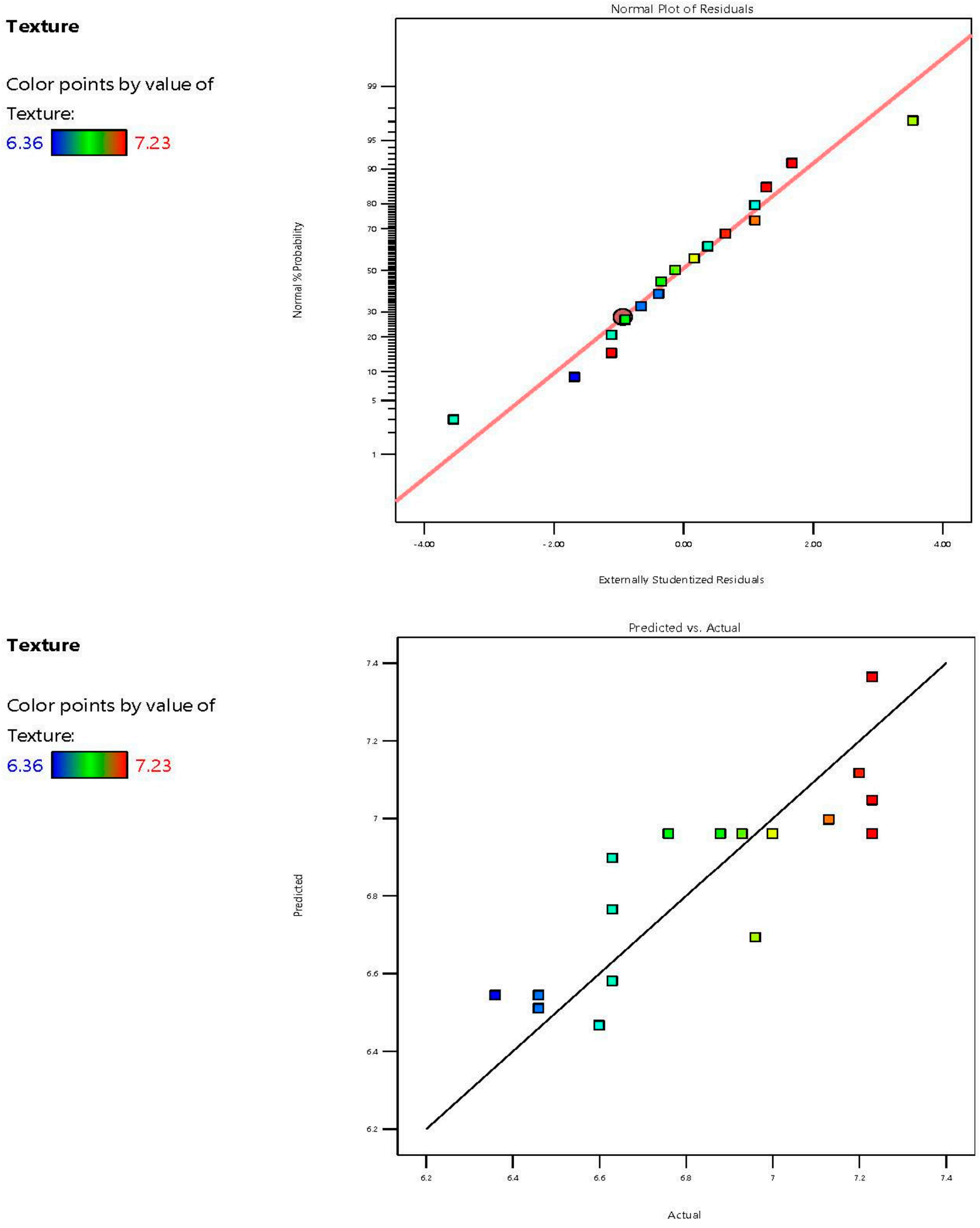
Texture
Color points by value of
Texture:
6.36
7.23
Normal Plot of Residuals
Normal % Probability
Externally Studentized Residuals
Texture
Color points by value of
Texture:
6.36
7.23
Predicted vs. Actual
Predicted
Actual

(a)

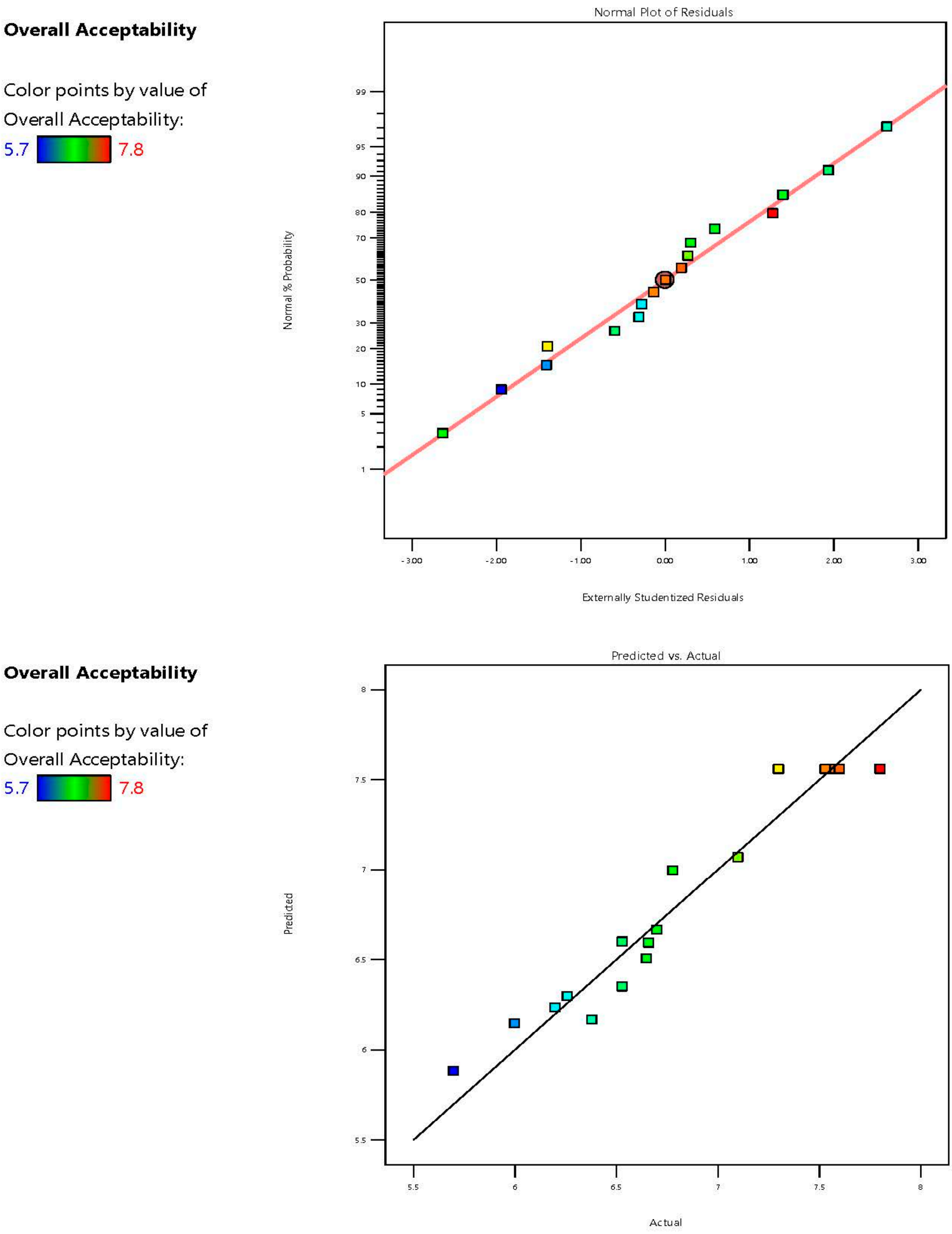

(b)

Figure S1. Normal plot of residual and predicted *vs.* actual. (a) color points by value of Texture; (b) color points by value of Overall Acceptability.
